# Pre-transplant bone marrow lymphocyte subsets predict outcome after allogeneic stem cell transplantation

**DOI:** 10.3389/fimmu.2026.1910059

**Published:** 2026-10-02

**Authors:** Stefan Koeck, Gabriele Hetzenauer, Josia Fauser, Verena Petzer, Sieghart Sopper, Ines Peschel-Schaar, Dominik Wolf, David Nachbaur, Normann Steiner

**Affiliations:** 1 Department of Internal Medicine V, Medical University of Innsbruck, Innsbruck, Tyrol, Austria; 2 Tyrolean Cancer Research Institute, Innsbruck, Austria; 3 Institute for Medical and Chemical Laboratory Diagnostics, Medical University of Innsbruck, Innsbruck, Austria

**Keywords:** allogenic stem cell transplantation, biomarker, flow cytometry, GvHD, lymphocyte subpopulations, predictive

## Abstract

**Introduction:**

Recent evidence has demonstrated that both donor- and recipient-derived immune cell subpopulations play a crucial role in various immune responses following allogeneic hematopoietic stem cell transplantation (HSCT). In this retrospective single center analysis, we investigated whether different lymphocyte subpopulations in the bone marrow (BM) of the recipient prior to HSCT might have an impact on outcome parameters after HSCT.

**Methods:**

BM aspirates of 140 patients > 18 years of age receiving a HSCT between 2008 and 2022 were analyzed. BM aspiration was performed within 3 months prior to HSCT. Median time from aspiration to HSCT was 14 days (range 8–104 days). Lymphocyte subpopulations included CD19^+^ B cells, CD56^+^ NK cells, CD3^+^ T cells, CD3^+^CD4^+^ T cells, CD3^+^CD8^+^ T cells, CD3^+^CD4^-^CD8^-^ T cells and CD3^+^CD4^+^CD8^+^ T cells.

**Results:**

High total lymphocytes > 37.35% (of leukocytes) correlated with an increased non-relapse mortality (NRM) at day 100 after HSCT, whereas high CD3^+^ T cells > 69.45% (of lymphocytes) and CD3^+^CD8^+^ T cells > 46.3% were associated with an increased risk for CMV infection. High NK cells > 8.8% resulted in an increased incidence for acute graft-versus host disease (GvHD). High CD19^+^ B cells > 13.1% resulted in a decreased incidence of chronic GvHD. In contrast, high CD4^+^ T cells > 49.3% were associated with an increased incidence of chronic GvHD.

**Conclusion:**

Recipient-derived lymphocyte subpopulations within the BM prior to HSCT comprise the prognostic potential to assess post-HSCT related outcome parameters. In summary, a shift towards humoral immunity with a BM characterized by a B cell enriched and T cell/NK cell depleted microenvironment appears to be associated with a lower risk for post-HSCT complications.

## 1 Introduction

Hematopoietic allogeneic stem cell transplantation (HSCT) represents a curative treatment option for various hematologic diseases (1), yet non-relapse mortality (NRM) and the risk of post-HSCT relapse alter the outcome of HSCT (2). Pre-transplant biomarkers are needed to estimate possible transplant-associated complications (3). However, current routine-based diagnostic options are not able to sufficiently predict outcome parameters such as overall survival (OS), relapse-free survival (RFS), NRM and the incidence of acute graft-versus-host disease (aGvHD) and chronic GvHD (cGvHD) (4, 5). Donor-derived immune cells are well-investigated, albeit recipient-derived immune cells are hardly understood regarding the impact on post-transplant outcome parameters. Immune cells surviving the conditioning treatment can shape the post-transplant immunological balance by modulating GvHD, the graft-versus-leukemia (GvL) effect and altering the pace and quality of immune reconstitution (5, 6).

Analyzing the composition of lymphocyte subpopulations within the peripheral blood and bone marrow (BM) niche is essential to illuminate the pathophysiological mechanisms underlying the GvL effect and the development of both aGvHD and cGvHD (6). Recent effort continues to investigate peripheral blood lymphocytes, yet the influence of BM derived lymphocytes subpopulations seems to be less examined. The BM comprises a specialized immunological niche that both generates and sustains adaptive lymphocytes whose phenotypic composition and functional states differ markedly from those of circulating cells (7, 8). Quantification and phenotypic analysis of B and T lymphocytes in bone marrow therefore provide a more accurate assessment of durable humoral and cellular immunity that cannot be inferred reliably from peripheral blood alone.

Given the central role of the BM as an immunological reservoir and its unique microenvironment, profiling lymphocyte subpopulations prior to HSCT may offer a more nuanced understanding of individual immunological predispositions. These insights might identify patients at higher risk for adverse outcomes and guide preemptive therapeutic strategies. In this retrospective study, we therefore analyzed specific lymphocyte subpopulations within the BM prior to HSCT and correlated their prognostic impact with key transplant-associated outcome parameters, including OS, RFS, NRM, cytomegalovirus (CMV) infection, and the incidence of aGvHD and cGvHD.

## 2 Methods

### 2.1 Study design and patient characteristics

In this retrospective study, adult patients who underwent HSCT between 2008 and 2022 were screened. Data of BM derived lymphocyte subpopulations were obtained together with patient related data including age, sex, diagnosis, date of transplant, type of donor (related/unrelated), conditioning regimen, GvHD prophylaxis, HLA mismatch, CMV status of patient and donor, remission status as well as date of death or date of last follow up (LFU), relapse status and cause of death (**Tables 1**, **2**).

**Table 1:** Patient characteristics.

Total = 140	n (%)	Total = 140	n (%)
Age median (range)	56 (21 - 76)	GvHD Prophylaxis n (%)	
Sex recipient		Calineurin Inhibitor + MTX	51 (36.4)
Female	62 (44.3)	Calineurin Inhibitor + MMF	87 (62.1)
Male	78 (55.7)	Calineurin Inhibitor + Steroid	1 (0.7)
Sex donor		No Immunosuppression	1 (0.7)
Female	51 (36.4)	ATG	15 (10.7)
Male	89 (63.6)	Conditioning	
Stem cell source		MAC	55 (39.3)
Peripheral blood	140 (100)	RIC	85 (60.7)
Bone marrow	0 (0)	Remission prior to HSCT	
Donor Type		Complete remission	86 (61.4)
MRD	54 (38.6)	Partial remission	10 (7.1)
MUD	62 (44.3)	Relapse/Refractory	35 (25.0)
MMUD	21 (15.0)	Unknown	9 (6.4)
Haploidentical	3 (2.1)	CMV status	
Diagnosis		Recipient pos/Donor pos	62 (44.3)
Acute leukemia	123 (87.9)	Recipient pos/Donor neg	37 (26.4)
MDS	5 (3.6)	Recipient neg/Donor pos	15 (10.7)
MPN	4 (2.9)	Recipient neg/Donor neg	26 (18.6)
Lymphoma	5 (3.6)		
CLL	2 (1.4)		
Aplastic Anemia	1 (0.7)		

ATG, anti-thymocyte globulin; CLL, chronic lymphocytic Leukemia; CMV, cytomegalovirus; CR, complete remission; GvHD, graft-versus-host disease; MAC, myeloablative conditioning; MDS, myelodysplastic Syndrome; MPN, myeloproliferative Neoplasia; MRD, matched related donor; MMF, mycophenolate mofetil; MMUD, mismatched related donor; MTX, methotrexate; MUD, matched unrelated donor; RIC, reduced intensity conditioning.

**Table 2:** Outcome parameter after allogeneic stem cell transplantation (HSCT).

Total = 140	n (%)	Total = 140	n (%)
Relapse after HSCT	50 (35.7)	Complications	
Death	76 (54.3)	Mucositis	76 (54.3)
Cause of Death		CMV infection	36 (25.7)
Relapse	34 (24.3)	Other infection	58 (41.4)
Non-Relapse	42 (30)	Bleeding	2 (1.4)
Median OS	2.3 years	Vascular complications	48 (34.3)
1-year OS	59.3	Acute GvHD	76 (54.3)
5-year OS	41.9	Chronic GvHD	48 (34.3)
Median RFS	n.r.		
1-year RFS	66.8		
5-year RFS	51.9		

CMV, cytomegalovirus; GvHD, graft-versus-host disease; n.r., not reached; OS, overall survival; RFS, relapse-free survival.

Outcome parameters included OS, RFS, NRM and the incidence of CMV infection, aGvHD and cGvHD. OS was defined as time from HSCT to death and RFS as time from HSCT to time point of relapse diagnosis. NRM was defined as death not caused by relapsed or progressive disease responsible for HSCT. CMV infection included CMV reactivation in seropositive recipients and primary infection in seronegative patients. CMV infection was defined as the detection of CMV in the peripheral blood by polymerase chain reaction (PCR) above the detection limit of 265 IU/ml or pp65 antigenemia with positivity of 2 out of 200000 cells, irrespective of the presence of clinical symptoms. CMV monitoring was performed weekly until day 100 after HSCT and subsequently every 2–4 weeks until day 365, with additional testing as clinically indicated. The first documented detection of CMV within 365 days after HSCT was considered the event. The diagnosis of aGvHD and cGvHD was determined based on clinical signs following the NIH guidelines (9, 10).

### 2.2 Ethical approval

This retrospective single center analysis has been approved by the ethics committee of the Medical University of Innsbruck (Nr. 1009/2023 and 1361/2022) and was conducted in accordance with the principles of the Declaration of Helsinki. Written informed consent for the use of the data was obtained in the context of the HSCT from each patient.

### 2.3 Bone marrow aspiration and flow cytometry

Bone marrow aspiration was performed within 3 months prior to HSCT. Median time from bone marrow aspiration to HSCT was 14 days (range 8–104 days). The first 3 to 5 milliliters (mL) of the aspiration were used for flow cytometry analysis to minimize dilution with peripheral blood and BM smears were morphologically assessed for adequate marrow representation. Predefined internal flow cytometry quality criteria, including evidence of sufficient erythropoiesis, were used to identify potentially hemodiluted samples. In addition, a bone marrow quality index (11) was retrospectively calculated where applicable to further assess sample quality and potential hemodilution. The index was applicable to 121 of 140 patients, of whom 118 (97.5%) had an index value >2.5, suggesting a low likelihood of clinically relevant hemodilution. Analysis of lymphocytes was performed by flow cytometry established within the clinical routine. Cell debris was excluded by SSC-A/FSC-A and doublets were excluded by FSC-H/FSC-A (**Supplementary Figure 1**). No specific viability/dead cell staining was used. Analyzed lymphocyte subpopulations included CD19^+^ B cells, CD3^-^CD56^+^ natural killer (NK) cells, CD3^+^ T cells, CD3^+^CD4^+^ T cells, CD3^+^CD8^+^ T cells, CD3^+^CD4^-^CD8^-^ T cells and CD3^+^CD4^+^CD8^+^ T cells. Results for CD3^+^CD4^-^CD8^-^ T cells and CD3^+^CD4^+^CD8^+^ T cells were available in 73 patients, all other subpopulations were measured in all patients (n = 140). All flow cytometry analyzes were performed by the certified Central Institute of Medical and Chemical Laboratory Diagnostics of the University Hospital of Innsbruck (certification: ISO 15189) without exception. To ensure longitudinal comparability of flow cytometric measurements over the entire study period (2008–2022), standardized instrument and staining controls were performed two to three times weekly in our accredited flow cytometry laboratory. Continuous monitoring of instrument performance enabled the detection of technical deviations and ensured consistent and reproducible analytical conditions throughout the study period. In total, 100 microliters (µl) of whole blood were stained with the appropriate fluorochrome-conjugated monoclonal antibodies at room temperature, protected from light. Before measuring cells on the flow cytometer, red blood cells were lysed (BD FACS Lysing Solution) and samples were washed and resuspended in phosphate buffered saline. Flow cytometry analyses were performed on the BD FACSCanto™ II (BD Biosciences, USA). All fluorochrome-conjugated monoclonal antibodies were obtained from BD Biosciences and can be found in the **Supplementary Table 1**. The panel included the following antibodies and respective clones: CD8-FITC (SK1), CD56-PE (MY31), CD4-PerCPCy5.5 (SK3), CD19-PE Cy7 (SJ25C1), CD3-APC (SK7), CD45-V450 (2D1). Gating strategy is shown in **Supplementary Figure 1**. Lymphocytes were calculated as percentage of CD45^+^ leukocytes, whereas lymphocytes subpopulations were presented as percentage of lymphocytes.

### 2.4 Statistical methods

Predefined lymphocyte subsets and clinically predefined patient subgroups were investigated. Tests for statistical significance were performed by using the Mann-Whitney U Test or T test for continuous variables and the Qui-Square Test. Kolmogorov–Smirnov test was used for testing of normal distribution. Cut-off values were determined with receiver operating characteristic (ROC) analyses and the optimal Youden-Index. For survival analysis, the log-rank test determined significance (p-value < 0.05) and Kaplan-Meier curves were obtained. Cox regression was used for the multivariate analyses. Spearman correlation was used for bivariate correlation. Internal validation of the ROC-derived cut-off values was performed using 2000 bootstrap resamples (**Supplementary Table 3**). SPSS Version 29 (IBM, USA) was used for statistical analyses. Competing risk analysis and bootstrapping were performed with RStudio (Posit PBC, USA, Version 2024.04.2). To account for multiple testing, each lymphocyte subpopulation was analyzed in relation to a specific clinical endpoint. When more than one endpoint was assessed for the same lymphocyte subpopulation, a Bonferroni correction was applied. Adjusted p-values were used to determine statistical significance. Regarding total lymphocytes, the overall cohort were corrected for three clinical endpoints (OS, NRM, and RFS). Subgroup analyses according to remission status and conditioning intensity for OS and NRM as well as patient at risk for CMV were considered exploratory.

## 3 Results

### 3.1 Patient characteristics

Patient characteristics and outcome parameters for the entire cohort are summarized in **Tables 1**, **2**. The median age was 56 years, 44.3% were female, and acute leukemia was the most frequent diagnosis. Donors were predominantly matched related (38.6%) or matched unrelated (44.3%), with fewer mismatched unrelated (15%) and haploidentical donors (2.1%). Reduced-intensity conditioning (RIC) was used in 60.7% of cases, while 39.3% received myeloablative conditioning (MAC). At transplantation, 61.4% of patients were in complete remission. CMV reactivation in seropositive recipients occurred in 16 of 37 patients (43%), whereas primary infection in seronegative patients was observed in 3 of 41 (7.3%). Median onset of CMV infection was 250 days after HSCT (range 56–2452 days). Acute GvHD of any grade occurred in 76 patients (54.3%) and cGvHD in 48 patients (34.3%). In 24 patients (17.1%) both aGvHD and cGvHD occurred, whereas in 40 patients (28.5%) no manifestation of GvHD, either acute or chronic, was observed. Median follow-up was 27.4 months (range 0.25 - 119.5 months).

The median proportion of lymphocytes among leukocytes was 13.15%. Within the lymphocyte compartment, T cells represented the predominant population with 83.4%, followed by NK cells and 9.5% and B cells with 1.7%. Among T-cell subsets, CD4+ T cells accounted for a median of 46.15%, CD8+ T cells for 30.95%, CD4+CD8+ T cells for 1.0%, and CD3^+^CD4^−^CD8^−^ T cells for 1.5%. All lymphocyte subsets showed a high variability of percentages (**Supplementary Table 2**).

### 3.2 Increased lymphocytes are associated with an increased early NRM

To explore the prognostic relevance of BM-derived lymphocyte subpopulations on early survival, optimal cut-off values for NRM were calculated. Most subsets did not yield significant associations with early mortality. However, total lymphocytes demonstrated a significant predictive value for NRM at day 100 (AUC = 0.692, p = 0.044; **Supplementary Figure 2**). Patients who died tended to have elevated lymphocyte percentages in the pre-transplant BM samples, although this trend did not reach statistical significance (**Figure 1A**). However, patients with lymphocyte percentages above the cut-off of 37.35% experienced a markedly worse early OS (58.8% vs 87.8%, adjusted p < 0.003, **Figure 1B**). Subgroup analyses revealed that the association between elevated lymphocyte percentages and poor OS was confined to patients undergoing RIC, with no significant effects observed in the MAC cohort (**Figure 1C**). Moreover, the prognostic effect was strongest in patients transplanted in complete remission (CR), highlighting a potential interaction between immune status and disease burden (**Figure 1D**).

**Figure 1:**
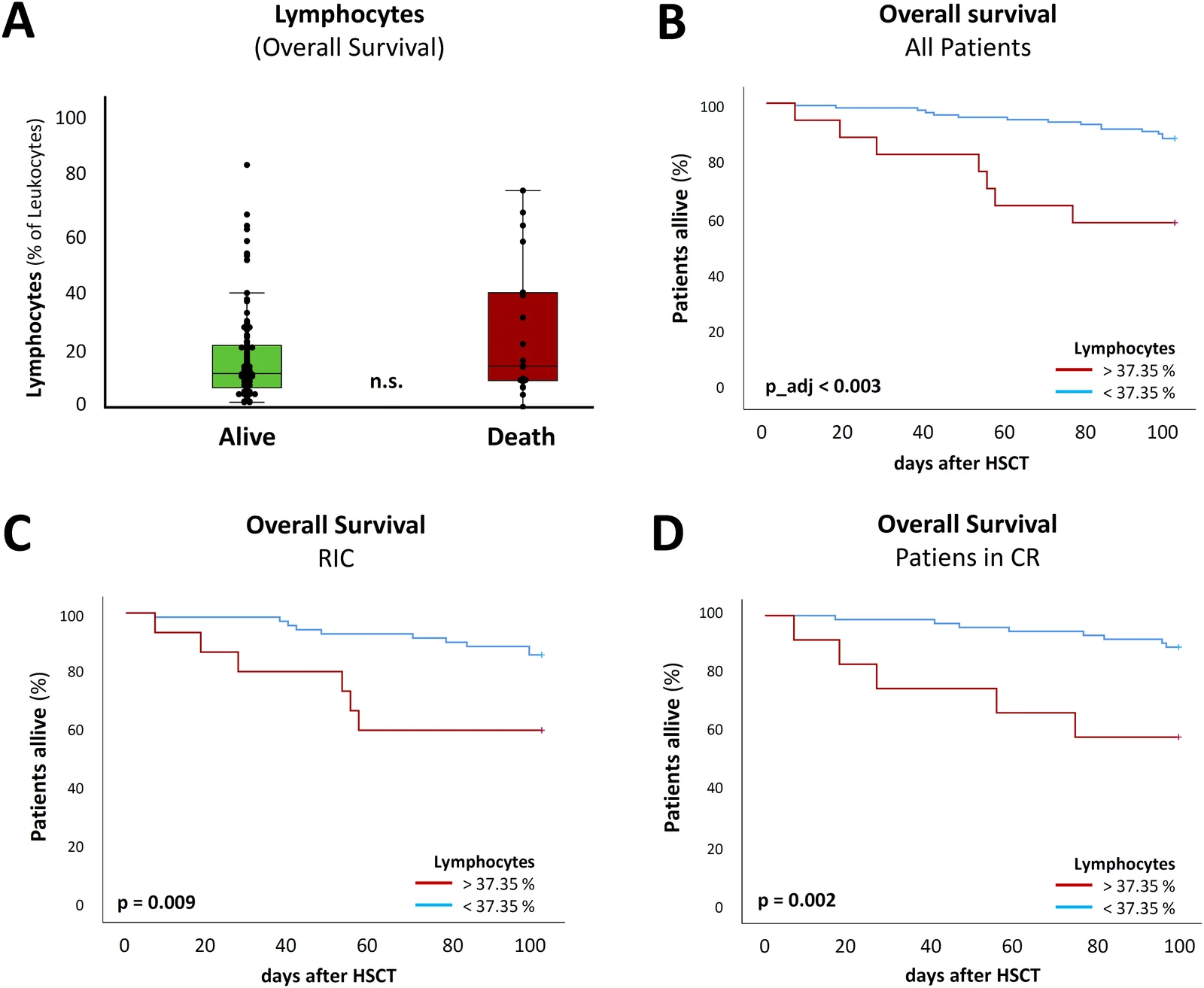
Association of pre-transplant bone marrow lymphocyte percentages with overall survival (OS) after allogeneic hematopoietic stem cell transplantation (HSCT). **(A)** Box plot showing bone marrow lymphocyte percentages in patients alive (green) and deceased (red). Kaplan–Meier plots show day-100 OS comparing high versus low pre-transplant bone marrow lymphocyte percentages for **(B)** the entire cohort, **(C)** patients receiving reduced-intensity conditioning (RIC) and **(D)** patients in complete remission (CR) at transplantation. Lymphocyte values are expressed as percentages of leukocytes. n.s. = not significant. p_adj = adjusted p-value with Bonferroni correction.

Regarding the NRM, patients who died from causes other than relapse also showed a tendency towards elevated lymphocyte percentages without statistical significance (**Figure 2A**), yet patients with lymphocyte percentages above the cut-off of 37.35% experienced a clearly increased NRM (4.1% vs. 29.9%, adjusted p = 0.006, **Figure 2B**). Competing risk analysis for NRM with relapse mortality as competing risk showed a hazard ratio (HR) of 7.1 (95% CI, 2.1 to 24.1, adjusted p = 0.0048) for elevated lymphocyte percentages. Regarding the subgroup analysis, differences in NRM were observed in patients undergoing RIC (**Figure 2C**) and patients in CR (**Figure 2D**). Competing risk analysis showed also significant results for both RIC patients (HR 8.1, 95% CI 2 to 33.3, adjusted p = 0.0117) and patients in CR (HR 6.6, 95% CI 1.7 to 25.7, adjusted p = 0.0189). Interestingly, RFS remained unaffected, suggesting that the observed impact is specific to NRM rather than disease recurrence. It is important to note that overall event numbers were low across OS, NRM, and RFS analyses, warranting cautious interpretation. Although CD3^+^CD4^+^CD8^+^ double positive T cells showed also significant results in the ROC analysis (**Supplementary Figure 2**), no significant differences were observed regarding the early day 100 OS and NRM.

**Figure 2:**
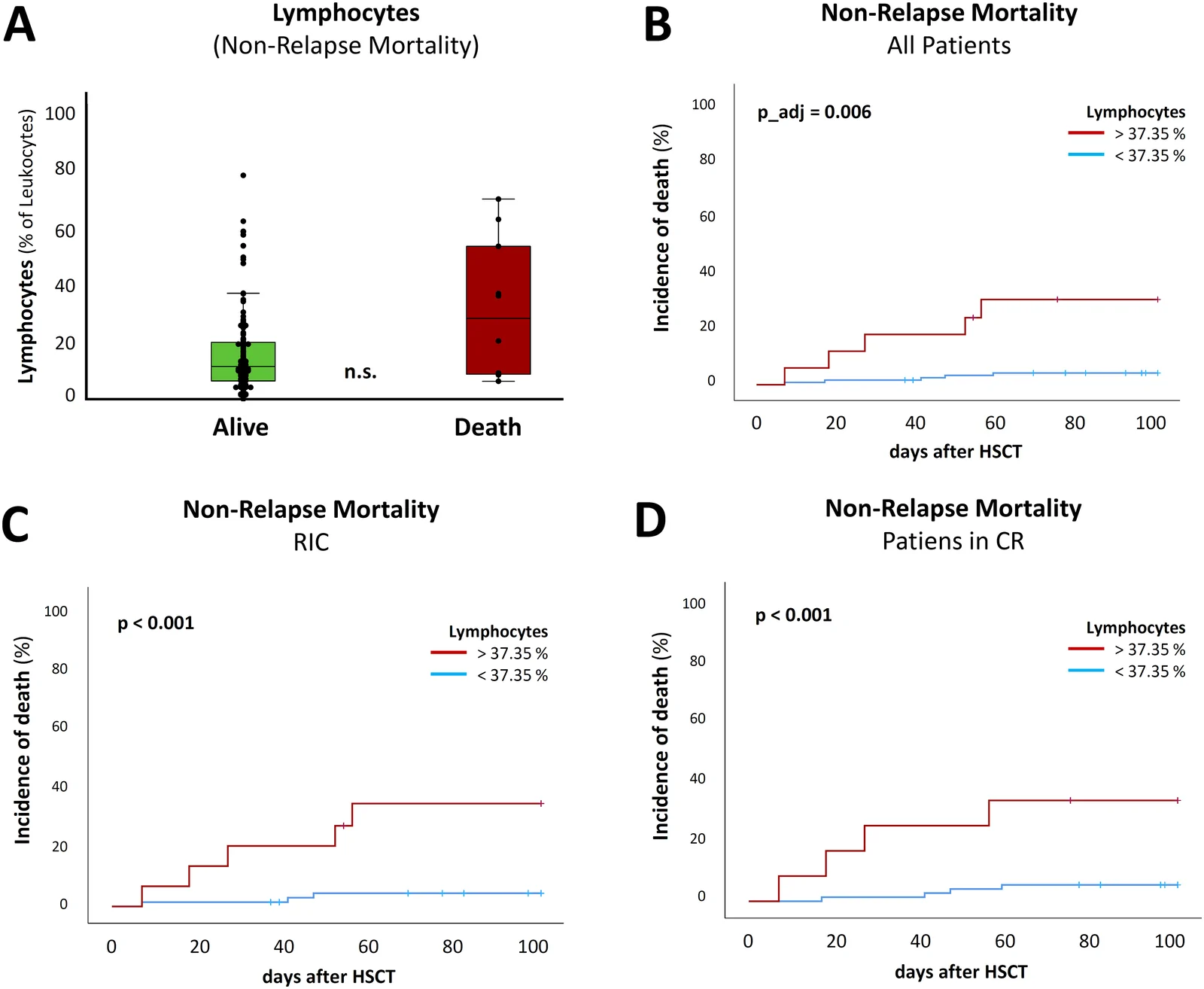
Association of pre-transplant bone marrow lymphocyte percentages with non-relapse mortality (NRM) after allogeneic hematopoietic stem cell transplantation (HSCT). **(A)** Box plot showing bone marrow lymphocyte percentages in patients alive (green) and deceased (red). Kaplan–Meier plots show day-100 NRM comparing high versus low pre-transplant bone marrow lymphocyte percentages for **(B)** the entire cohort, **(C)** patients receiving reduced-intensity conditioning (RIC) and **(D)** patients in complete remission (CR) at transplantation. Lymphocyte values are expressed as percentages of leukocytes. n.s. = not significant. p_adj = adjusted p-value with Bonferroni correction.

### 3.3 Increased CD3^+^CD8^+^ T cells predict CMV infection in the first year after HSCT

Pre-transplant BM lymphocyte subsets were analyzed for the association with CMV infection within one year after HSCT. No differences were observed in total lymphocytes, NK cells, CD19^+^ B cells, or CD4^+^ T cell subsets between patients with and without CMV infection. In contrast, patients who developed CMV infection exhibited significantly higher percentages of total CD3^+^ T cells (p = 0.002) and CD3^+^CD8^+^ T cells (p = 0.001) prior to HSCT (**Figures 3A, B**), and cut-off analyses confirmed their predictive value (**Supplementary Figure 3**). Elevated CD3^+^ T cells > 69.45% and CD3^+^CD8^+^ T cells > 46.3% were associated with earlier onset and higher incidence of CMV infection. The 1-year cumulative incidence of CMV infection was 27.4% vs. 0% for high vs. low CD3^+^ proportions (p = 0.009, **Figure 3C**) and 52.2% vs. 16.2% for high vs. low CD3^+^CD8^+^ proportions (p < 0.001; **Figure 3D**). These associations remained significant in patients at high-risk for CMV infection, defined by a CMV-seropositive recipient status (**Figures 3E, F**). Competing risk analysis was not applicable for CD3^+^ T cells due to perfect separation. Regarding CD3^+^CD8^+^ T cells, competing risk analysis with death as competing risk showed a HR of 5.3 (95% CI 2.2 to 12.7, p = 0.0002) for all patients and a HR of 6.4 (95% CI 2.3 to 18.3, p = 0.0005) for high-risk patients.

**Figure 3:**
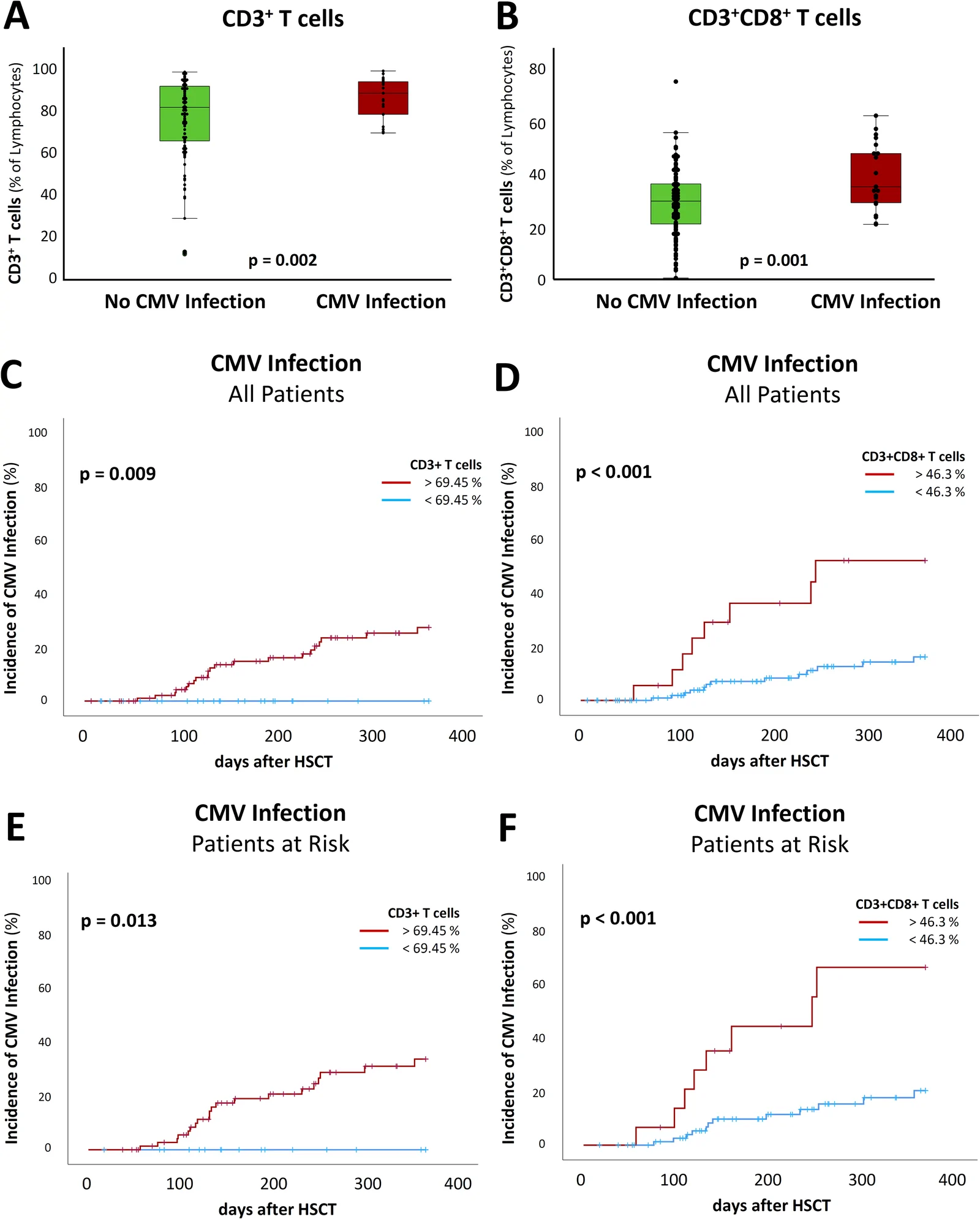
Association of pre-transplant bone marrow CD3^+^ T cell and CD3^+^CD8^+^ T cell percentages with the incidence of cytomegalovirus (CMV) infection after allogeneic hematopoietic stem cell transplantation (HSCT). Box plot showing **(A)** bone marrow CD3^+^ T cell percentages and **(B)** CD3^+^CD8^+^ T cell percentages in patients without CMV infection (green) and confirmed CMV infection (red). Kaplan-Meier plots show the cumulative incidence of CMV infection in the first year after HSCT for **(C, D)** all patients and **(E, F)** patients at risk comparing high versus low pre-transplant percentages of CD3^+^ T cells and CD3^+^CD8^+^ T cells. Patients at risk were defined as CMV seropositive status of the recipient. CD3^+^ T cell and CD3^+^CD8^+^ T cell values are expressed as percentages of lymphocytes.

### 3.4 Pre-transplant BM lymphocyte subsets differentially predict acute and chronic GvHD

To identify immunological predictors of GvHD, we systematically analyzed all pre-transplant BM lymphocyte subpopulations in relation to both aGvHD and cGvHD. The majority of lymphocyte subsets showed no significant association with both outcome parameters, but NK cells and CD3^+^CD4^-^CD8^-^ T cells correlated with an altered risk for aGvHD, and CD19^+^ B cells and CD3^+^CD4^+^ T helper cells showed an association with cGvHD (**Figures 4**, **5**).

**Figure 4:**
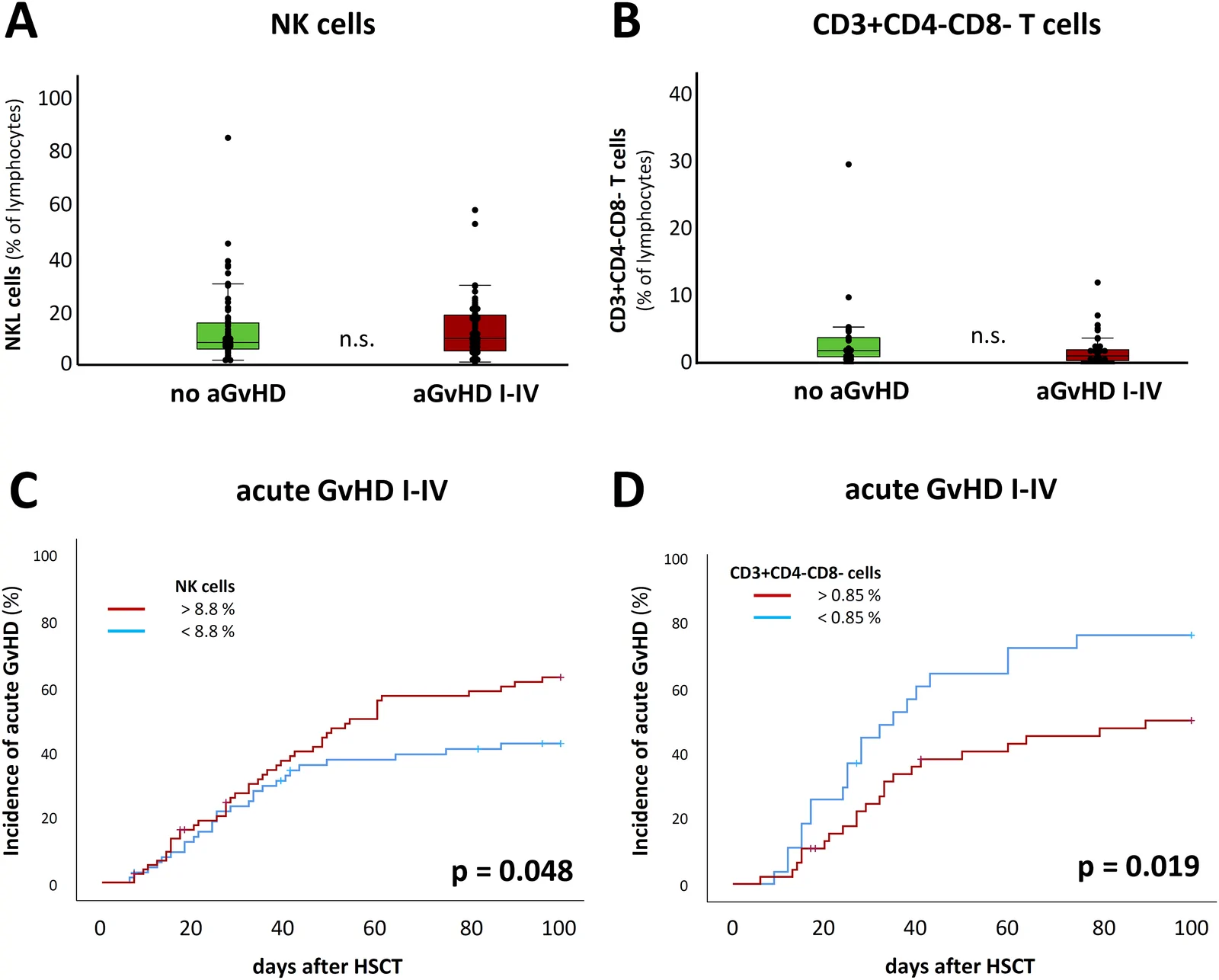
Association of pre-transplant bone marrow NK cell and CD3^+^CD4^−^CD8^−^ T cell percentages with the incidence of acute graft-versus-host disease (GvHD) after allogeneic hematopoietic stem cell transplantation (HSCT). Box plots show **(A)** bone marrow NK cell percentages and **(B)** CD3^+^CD4^−^CD8^−^ T cell percentages in patients without acute GvHD (green) and with acute GvHD grade I-IV (red). Kaplan-Meier plots show the cumulative incidence of acute GvHD I-IV after HSCT comparing high versus low pre-transplant percentages of **(C)** NK cells and **(D)** CD3^+^CD4^−^CD8^−^ T cells. NK cell and CD3^+^CD4^−^CD8^−^ T cell values are expressed as percentages of lymphocytes. n.s. = not significant.

**Figure 5:**
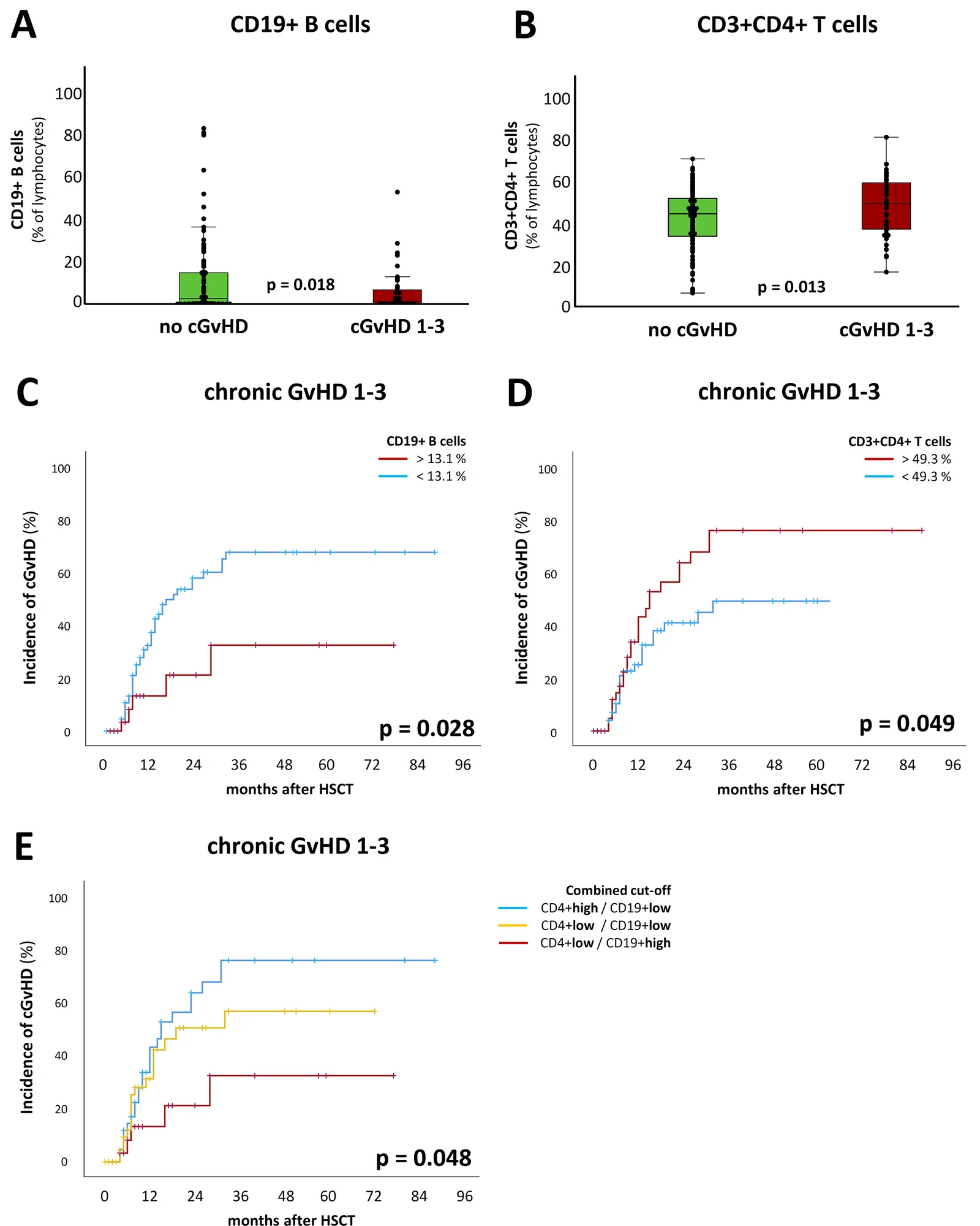
Association of pre-transplant bone CD3^+^CD4^+^ T cell and CD19^+^ B cell percentages with the incidence of chronic graft-versus-host disease (GvHD) after allogeneic hematopoietic stem cell transplantation (HSCT). Box plots show **(A)** bone marrow CD3^+^CD4^+^ T cell percentages and **(B)** CD19^+^ B cell percentages in patients without chronic GvHD (green) and with chronic GvHD grade 1-3 (red). Kaplan-Meier plots show the cumulative incidence of chronic GvHD 1–3 after HSCT comparing high versus low pre-transplant percentages of **(C)** CD3^+^CD4^+^ T cells, **(D)** CD3^+^CD4^+^ T cells and **(E)** combined cut-off values to categorize patients into 3 groups: CD4^+high^CD19^+low^, CD4^+low^CD19^+high^ and CD4^+low^CD19^+low^. No patients expressed a CD4^+high^CD19^+high^ profile. CD3^+^CD4^+^ T cell and CD19^+^ B cell values are expressed as percentages of lymphocytes. n.s. = not significant.

For aGvHD, comparison of patients with and without aGvHD showed no differences in NK cell and CD3^+^CD4^-^CD8^-^ T cell percentages (**Figures 4A, B**). NK-cell and CD3^+^CD4^-^CD8^-^ T-cell percentages were not correlated (Spearman’s ρ = −0.075, *p* = 0.530, **Supplementary Figure 4**). ROC curve analysis identified CD3^+^CD4^-^CD8^-^ T cells as significantly associated with aGvHD risk although the association was weak, whereas NK cells did not reach significance (**Supplementary Figure 5**). Moreover, for NK cells bootstrap validation showed a different optimal cut-off compared with the initially derived threshold (**Supplementary Table 3**). Nevertheless, survival analysis demonstrated that both populations were associated with aGvHD-free survival. Patients with NK cell percentages >8.8% experienced a significantly higher incidence of aGvHD (63.0% vs. 42.7%, p = 0.048; **Figure 4C**), whereas elevated CD3^+^CD4^-^CD8^-^ T-cell percentages >0.85% were associated with a reduced incidence of aGvHD (50.2% vs. 76.4%, p = 0.019; **Figure 4D**). Competing risk analysis with death as competing risk revealed a significant difference for CD3^+^CD4^-^CD8^-^ T cells (HR 0.49, 95% CI 0.27 to 0.89, p = 0.018), but not for NK cells (HR 1.6, 95% CI 1 to 2.5, p = 0.068). CD19^+^ B cells and CD3^+^CD4^+^ T helper cells were not associated with aGvHD.

In contrast, cGvHD was associated with a different immunological profile. Patients who developed cGvHD had significantly lower percentages of CD19^+^ B cells (5.4% vs. 11.1%, p = 0.018; **Figure 5A**) and higher percentages of CD3^+^CD4^+^ T helper cells (48.5% vs. 42.1%, p = 0.013; **Figure 5B**). These two populations showed a moderate inverse correlation (Spearman’s ρ = −0.499, p < 0.001; **Supplementary Figure 6**). ROC curve analysis reached significance only for CD4^+^ T cells (**Supplementary Figure 7**). Survival analysis revealed that low CD19^+^ B cell percentages were associated with a significantly higher and earlier onset of cGvHD (1-year and 2-year incidence: 32.2% and 57.7% vs. 14% and 21.2%, p = 0.028, **Figure 5C**). Conversely, lower CD4^+^ T cell counts correlated with a delayed and reduced incidence of cGvHD compared to patients with elevated CD4^+^ levels (1-year: 25% vs. 33.6%; 2-year: 41% vs. 63.5%, p = 0.049; **Figure 5D**). Competing risk analysis with death as competing risk showed a significant reduced risk for increased CD19^+^ B cells (HR 0.37, 95% CI 0.15 to 3, p = 0.035) and increased risk for CD3^+^CD4^+^ T cells (HR 1.7, 95% CI 1 to 3, p = 0.046). NK cells and CD3^+^CD4^-^CD8^-^ T cells were not associated with cGvHD.

To further investigate the prognostic impact of these two immune compartments, we performed a combined analysis based on cut-off values. Three distinct immunological profiles emerged: CD4^+^_high_CD19^+^_low_, CD4^+^_low_CD19^+^_high_, and CD4^+^_low_CD19^+^_low_. Notably, no patients exhibited a CD4^+^_high_CD19^+^_high_ profile. The CD4^+^_high_CD19^+^_low_ group showed the highest cGvHD incidence (1-year: 33.6%, 2-year: 63.5%), while the CD4^+^_low_CD19^+^_high_ group had the lowest (1-year: 13.3%, 2-year: 21.2%). The CD4^+^_low_CD19^+^_low_ group displayed intermediate risk (1-year: 31.2%, 2-year: 50.4%; **Figure 5E**). Therefore, the CD4^+^_high_CD19^+^_low_ constellation represents a potential high-risk immunological signature for cGvHD.

### 3.5 Multivariate analysis

To assess the independent prognostic value of BM-derived lymphocyte subsets, multivariate analyses including relevant clinical covariates were performed. Multivariate analyses adjusting for clinical covariates identified total pre-HSCT BM-derived lymphocytes as an independent predictor of early outcome after HSCT. Elevated lymphocyte proportions were associated with increased mortality within 100 days, affecting both OS (HR 3.4, 95% CI 1.3 - 9.2, p = 0.015) and NRM (HR 6.4, 95% CI 1.6 - 25.9, p = 0.008), whereas established clinical risk factors were not significant (**Table 3**). Increased CD3^+^CD8^+^ T cell levels independently predicted CMV infection (HR 5.0, 95% CI 1.9 - 13.1, p = 0.001), whereas CD3^+^ T cells were not significant in multivariate models. Regarding aGvHD, higher CD56^+^ NK cell proportions showed no increased risk (HR 1.4, 95% CI 0.9-2.3, p = 0. 179), while CD4^-^CD8^-^ T cells were independently protective against aGvHD (HR 0.4, 95% CI 0.2-0.9, p = 0.0015). The year of HSCT was identified as significant co-factor for CD3^+^CD8^+^ T cells and NK cells (**Table 4**). In cGvHD analyses, CD19^+^ B cells were associated with a protective effect (HR 0.4, 95% CI 0.2-1.0, p = 0.062), while higher CD4^+^ T cell proportions conferred increased risk (HR 1.6, 95% CI 0.9-3.0, p = 0.115). However, both multivariate analyses only showed a tendency towards significance. A combined immunophenotype of high CD19^+^ B cell and low CD3^+^CD4^+^ T cell percentages showed no statistical significance. Donor mismatch emerged as significant co-factor for CD19^+^ B cells (**Table 4**).

**Table 3:** Multivariate analysis of statistically significant lymphocyte subpopulations for OS, NRM and CMV infection.

	OS		NRM		CMV Infection	
	n = 140, events = 22		n = 140, events = 10		n = 140, events = 21	
Variable	HR (95% CI)	p	HR (95% CI)	p	HR (95% CI)	p
Lymphocytes						
> 37.35%	3.4 (1.3 - 9.2)	0.015	6.4 (1.6 - 25.6)	0.008		
CD3+CD8+ T cells						
> 46.3%					5.0 (1.9 - 13.1)	0.001
Mismatch Donor	1.5 (0.5 - 4.6)	0.500	0.4 (0.0 - 3.4)	0.394	2.7 (0.7 - 10.1)	0.139
RIC vs. MAC	2.1 (0.6 - 7.0)	0.232	2.4 (0.3 - 16.6)	0.378	3.6 (0.9 - 14.7)	0.069
Non-CR vs. CR	1.2 (0.5 - 3.0)	0.698	0.5 (0.1 - 2.4)	0.370	1.3 (0.5 - 3.3)	0.594
ATG vs. no ATG	0.9 (0.2 - 3.6)	0.849	2.8 (0.3 - 23.6)	0.332	0.9 (0.2 - 4.4)	0.854
< 55y vs. > 55y	0.6 (0.2 - 1.7)	0.337	0.6 (0.2 - 2.7)	0.554	1.3 (0.4 - 4.3)	0.671
Year of HSCT	1.0 (0.8 - 1.2)	0.772	0.9 (0.7 - 1.3)	0.716	0.8 (0.6 - 1.0)	0.028

Multivariate analysis was performed with cox regression model using the defined cut-off parameter and the respective co-variates. ATG, anti-thymocyte globulin; CI, confidence interval; CR, complete response; HR, hazard ratio; HSCT, hematopoietic stem cell transplantation; MAC, myeloablative conditioning; NRM= non-relapse mortality; OS, overall survival; RIC, reduced intensity conditioning; y, years.

**Table 4:** Multivariate analysis of statistically significant lymphocyte subpopulations for acute (aGvHD) and chronic (cGvHD) graft-versus-host disease.

	Acute GvHD		Acute GvHD			
	n = 140, events = 72		n = 73, events = 42			
Variable	HR (95% CI)	P	HR (95% CI)	p		
*CD56+ NK cells*						
> 8.8%	1.4 (0.9 - 2.3)	0.179				
*CD4-CD8- T cells*						
> 0.85%			0.4 (0.2 - 0.9)	0.015		
Mismatch Donor	2.6 (1.3 - 5.2)	0.008	4.3 (1.3 - 14.0)	0.017		
RIC vs. MAC	0.6 (0.3 - 1.1)	0.08	0.5 (0.2 - 1.3)	0.144		
Non-CR vs. CR	1.9 (1.2 - 3.1)	0.009	2.3 (1.2 - 4.5)	0.01		
ATG vs. no ATG	0.5 (0.2 - 1.3)	0.155	0.4 (0.1 - 1.9)	0.221		
< 55y vs. > 55y	2.2 (1.1 - 4.3)	0.018	2.8 (1.1 - 7.0)	0.032		
Year of HSCT	1.1 (1.0 - 1.3)	0.032	0.9 (0.7 - 1.2)	0.614		

	Chronic GvHD		Chronic GvHD		Chronic GvHD	
	n = 140, events = 48		n = 140, events = 48		n = 140, events = 48	
	HR (95% CI)	P	HR (95% CI)	p	HR (95% CI)	p
*CD19+ B cells*						
> 13.1%	0.4 (0.1 - 1.0)	0.062				
*CD4+ T cells*						
> 49.3%			1.6 (0.9 - 3.0)	0.115		
*Combined*						
CD19+^high^/CD4+^low^					0.9 (0.6 - 1.2)	0.369
Mismatch Donor	2.8 (1.2 - 7.0)	0.024	2.3 (1.0 - 5.6)	0.06	2.3 (0.9 - 5.5)	0.07
RIC vs. MAC	2.1 (0.9 - 5.1)	0.102	2.2 (0.9 - 5.3)	0.077	2.3 (1.0 - 5.5)	0.065
Non-CR vs. CR	1.1 (0.6 - 2.2)	0.687	1.2 (0.6 - 2.4)	0.581	1.1 (0.6 - 2.2)	0.742
ATG vs. no ATG	0.6 (0.2 - 2.2)	0.475	0.7 (0.2 - 2.5)	0.599	0.7 (0.2 - 2.5)	0.629
< 55y vs. > 55y	1.6 (0.8 - 3.5)	0.217	1.7 (0.8 - 3.7)	0.171	1.7 (0.8 - 3.7)	0.156
Year of HSCT	0.9 (0.8 - 1.1)	0.199	0.9 (0.8 - 1.1)	0.403	0.9 (0.8 - 1.1)	0.352

Multivariate analysis was performed with cox regression model using the defined cut-off parameter and the respective co-variates. ATG, anti-thymocyte globulin; CI, confidence interval; CR, complete response; HR, hazard ratio; HSCT, hematopoietic stem cell transplantation; MAC, myeloablative conditioning; NRM, non-relapse mortality; OS, overall survival; RIC, reduced intensity conditioning; y, years.

The number of events were low, particularly for NRM (OS 22 events, NRM 10 events, CMV infection 21 events, aGvHD 72 and cGvHD 48). To assess the potential for overfitting in the multivariable analyses, reduced cox regression models including the specific lymphocyte subpopulation and two of the clinically important co-variates were performed (**Supplementary Table 4**). For OS, NRM and CMV infection, the association remained statistically significant and HR were comparable. For aGvHD, HR were comparable and in addition the results for NK cells were significant compared to the original model. For cGvHD, HR remained similar to the original model, but results for CD19^+^ B cells were significant and showed a borderline significance for CD4^+^ T cells.

## 4 Discussion

Recipient-derived immune cells surviving the HSCT influence significantly post-transplant immune responses by interfering with the novel donor-derived immune system (12, 13). Most previous studies have focused on peripheral blood samples or murine models, whereas lymphocytes residing in the BM have received comparatively less attention, although the BM niche comprises a pivotal role in the development and severity of HSCT complications such as GvHD (14, 15).

In our analysis, elevated percentages of total lymphocytes in the BM prior to HSCT are associated with decreased OS within 100 days post-HSCT, primarily attributable to higher NRM. Our findings are consistent with previous studies investigating peripheral blood lymphocytes, which also reported an association of elevated lymphocytes and a reduced survival (16). Notably, these associations were less pronounced in patients receiving MAC and in those not in CR at transplantation. This finding may indicate that the prognostic relevance of pre-transplant BM immune composition differs according to conditioning intensity and disease status. In patients undergoing RIC and those in CR prior to transplantation, baseline immune characteristics may provide additional prognostic information, whereas their relevance may be less apparent in the presence of more pronounced treatment- or disease-related factors. In patients with active disease at transplantation, established disease-related prognostic markers, including measurable residual disease (MRD), may therefore limit the incremental prognostic value of pre-transplant immune profiling. The assessment of immune composition may provide complementary information alongside established disease-related biomarkers. Regarding CMV infection, our data showed an association between elevated percentages of CD3+ T cells, particularly CD3+CD8+ T cells, within the BM prior to HSCT on the occurrence of CMV infection within the first year after transplantation. These findings are particularly observed in patients at risk for CMV infection. Although post-HSCT immune reconstitution of T cells, especially CMV specific CD4^+^ and CD8^+^ T cells, was identified as a protective condition against CMV reactivation (17), we are able to show that recipient-derived CD8^+^ T cells within the BM comprise a prognostic value for the risk of CMV infection within the first year after HSCT.

Increased recipient-derived NK cells within the BM prior to HSCT are associated with the development of aGvHD, although statistical deviations limit the interpretation. Although previous studies have suggested that donor-derived NK cells may be associated with reduced GvHD incidence (18), recent investigations demonstrated that specific NK cell subsets are distributed differently within several organs, with a predominance of activated CD16^+^ NK cells within the BM (19). These observations support the relevance of the BM niche when interpreting the potential association of NK cells and post-transplant outcomes. Whether recipient-derived NK cells persist after transplantation and contribute directly to the observed association with aGvHD must be investigated in further studies. In contrast, recipient-derived double negative T cells within the BM prior to HSCT are associated with a reduced risk for aGvHD, consistent with observations regarding donor-derived double-negative T cells (20, 21). In summary, high NK cells and low double negative T cells within the BM prior to HSCT may be associated with an increased risk for aGvHD risk. These observations require validation in independent cohorts and functional studies.

Elevated CD19^+^ B cell levels correlate with a reduced incidence of cGvHD, indicating a potential association between recipient-derived B-cell abundance and a reduced risk for cGvHD. While donor-derived CD19^+^ B cells within the peripheral blood are associated with cGvHD (22), murine models have shown that recipient-derived CD19^+^ B cells are not essential for GvHD induction (23). Previous studies have demonstrated that activated B cells can modulate T-cell responses (23), providing a potential explanation for the observed association (24). In contrast, we are able to show that high percentages of CD4^+^ T helper cells in the BM prior to HSCT are associated with an increased incidence of cGvHD. T cells, particularly CD4^+^ subsets, have been implicated in GvHD development, and experimental depletion of CD4+ T cells has been associated with reduced GvHD severity in murine models (25). Our findings are also consistent with previous observations that CD4^+^CD25^-^ T helper cells, unlike the regulatory CD4^+^CD25^+^ population, are associated with an increased cGvHD risk (26). This is in line with studies indicating that depletion of CD4^+^ T cells enhances B cell recovery and mitigates GvHD (24). Since the majority of CD4^+^ T helper cells are non-regulatory and contribute to the activation of CD8^+^ cytotoxic T cells, their abundance in the BM may promote the immunological conditions that facilitate cGvHD onset (27). A combined analysis of CD4^+^ T cell and B cell percentages in the BM prior to HSCT revealed that high CD4^+^ with low B cell percentages are associated with the highest risk for developing cGvHD. In contrast, low CD4^+^ and high B cell levels correlated with the lowest risk. These findings suggest that a pre-HSCT BM microenvironment enriched in B cells and depleted in CD4^+^ T cells may present the most favorable prognostic factor for a reduced cGvHD. Although the associations of CD19^+^ B cells and CD3^+^CD4^+^ T cells with cGvHD were attenuated after multivariate adjustment, their direction and effect estimates remained consistent with the univariate analyses. This attenuation may reflect confounding by established clinical risk factors and/or limited statistical power due to the number of cGvHD events. These findings should therefore be regarded as exploratory and hypothesis-generating.

Our study has limitations, primarily due to its descriptive and retrospective design and single center recruitment. Future research should aim to elucidate the mechanism of our findings and further characterize the involved lymphocyte subpopulations in greater detail. Although we provided clinically informative stratification within our cohort, their retrospective-driven derivation remains a limiting factor and requires confirmation in independent and prospective patient cohorts. Furthermore, the BM puncture itself represents a technical limitation, as potential dilution of the BM aspirate with peripheral blood might impair the interpretation of lymphocyte subpopulations. Although the quality criteria indicated a high likelihood of adequate BM representation in the vast majority of evaluable samples, we did not exclude the few samples with questionable hemodilution to avoid a *post-hoc* selection bias in our retrospective study. Moreover, the lack of a healthy bone marrow reference cohort represents another important limitation of this study. Another restriction is the absence of a viability dye in the flow cytometry panel. Future investigations including healthy donor bone marrow or validated external reference datasets and the application of a viability staining can support the interpretation of our results. Several statistical limitations should be considered. Despite Bonferroni correction for analyses involving multiple endpoints, the number of lymphocyte subsets and endpoints may have increased the risk of chance findings. In addition, ROC-derived cut-offs were internally derived and the relatively small cohort with limited event numbers may have resulted in reduced power and potential overfitting of multivariable models, although reduced cox regression models showed comparable results. The findings should be considered exploratory and require validation in larger, independent cohorts.

Nevertheless, our findings indicate that the composition of recipient-derived lymphocyte populations in the BM prior to HSCT is associated with post-transplant outcomes. A shift toward humoral immunity with a BM characterized by a B cell enriched and T cell/NK cell depleted microenvironment appears to be associated with a lower risk of both aGvHD and cGvHD, as well as decreased likelihood of CMV reactivation. These observations suggest that the BM immune composition prior to HSCT might serve as a prognostic tool for post-transplant outcomes and should be explored in future interventional and mechanistic studies.

## Data Availability

The data that support the findings of this study are available from the corresponding author upon reasonable request.
