# Supplementary material for: Pre-transplant bone marrow lymphocyte subsets predict outcome after allogeneic stem cell transplantation
Source: Front Immunol. 2026 Oct 2;17:1910059. doi: 10.3389/fimmu.2026.1910059 (PMC13672203; doi:10.3389/fimmu.2026.1910059)
Supplement: Supplementary file 1 [file SupplementaryFile1.docx]

Supplementary Material

# 1 Supplementary Figures and Tables

| **Antibody** | **Vendor** | **LOT-Number** | **Flourochrome** |
| --- | --- | --- | --- |
| CD8-FITC (SK1) | BD Biosciences | 345772 | FITC |
| CD4-PerCPCy5.5 (SK3) | BD Biosciences | 332772 | PerCPCy5.5 |
| CD3-APC (SK7) | BD Biosciences | 345767 | APC |
| CD56-PE (MY31) | BD Biosciences | 345810 | PE |
| CD19-PECy7 (SJ5C1) | BD Biosciences | 341113 | PECy7 |
| CD45-V500 (2D1) | BD Biosciences | 655873 | V500 |

**Supplementary Table 1**: Information about the utilized flourochrome-conjugated monoclonal antibodies. All antibodies were obtained from BD Biosciences (USA).

|  | **Median (range) %** |
| --- | --- |
| **Lymphocytes** | 13.15 (0.4 - 84.3) |
| **B cells** | 1.7 (0 - 83.3) |
| **NK cells** | 9.5 (0.9 - 87.8) |
| **T cells** | 83.4 (12.3 - 98.9) |
| **CD4+ T cells** | 46.15 (6.6 - 81.5) |
| **CD8+ T cells** | 30.95 (0.8 - 75.1) |
| **CD4+CD+8 T cells** | 1 (0.1 - 10.6) |
| **CD4-CD8- T cells** | 1.5 (0 - 30.8) |

**Supplementary Table 2**: Percentages of bone marrow lymphocyte subsets of the entire cohort

|  |  | **Original** | **Bootstrapping Control** |
| --- | --- | --- | --- |
|  |  | Cut-off | Median |
| **Lymphocytes** | **NRM** | 37.5% | 38,05% |
| **CD3+** | **CMV** | 69.45% | 69.45% |
| **CD3+CD8+** | **CMV** | 46.3% | 33.65% |
| **NK** | **aGvHD** | 49.35% | 8.75% |
| **CD3+CD4-CD8-** | **aGvHD** | 0.85% | 0.85% |
| **CD19+** | **cGvHD** | 13.1% | 13.1% |
| **CD4+** | **cGvHD** | 49.35 % | 49.35 % |

**Supplementary Table 3**: Bootstrapping control of cut-off values

Internal validation of the ROC-derived cut-off values was performed using 2000 bootstrap resamples. The stability of the Youden-derived cut-off was assessed by estimating its bootstrap distribution. aGvHD = acute graft-versus-host disease, cGvHD = chronic graft-versus-host disease, CMV = cytomegalovirus, NRM = non-relapse mortality

|  | **OS** | | **NRM** | | **CMV Infection** | |
| --- | --- | --- | --- | --- | --- | --- |
|  | n = 140, events = 22 | | n = 140, events = 10 | | n = 140, events = 21 | |
| Variable | HR (95% CI) | p | HR (95% CI) | p | HR (95% CI) | p |
| ***Lymphocytes*** |  |  |  |  |  |  |
| **> 37.35%** | 4.0 (1.6 - 10.3) | 0.004 | 6.7 (1.8 - 24.9) | 0.004 |  |  |
| **CD3+CD8+ T cells** |  |  |  |  |  |  |
| **> 46.3%** |  |  |  |  | 4.1 (1.7 - 10.1) | 0.002 |
| Mismatch Donor |  |  |  |  |  |  |
| RIC vs. MAC | 1.4 (0.5 - 3.8) | 0.480 | 1.7 (0.3 - 8.8) | 0.519 | 2.6 (0.9 - 7.3) | 0.075 |
| Non-CR vs. CR | 1.2 (0.5 - 2.8) | 0.686 | 0.5 (0.1 - 2.1) | 0.318 |  |  |
| ATG vs. no ATG |  |  |  |  | 1.5 (0.4 - 5.5) | 0.503 |
|  | **Acute GvHD** | | **Acute GvHD** | |  |  |
|  | n = 140, events = 72 | | n = 73, events = 42 | |  |  |
| Variable | HR (95% CI) | p | HR (95% CI) | p |  |  |
| ***CD56+ NK cells*** |  |  |  |  |  |  |
| **> 8.8%** | 1.7 (1.0 - 2.7) | 0.035 |  |  |  |  |
| ***CD4-CD8- T cells*** |  |  |  |  |  |  |
| **> 0.85%** |  |  | 0.5 (0.2 - 0.9) | 0.017 |  |  |
| Mismatch Donor | 2.3 (1.2 - 4.5) | 0.015 | 3.1 (1.0 - 10.1) | 0.058 |  |  |
| ATG vs. no ATG | 0.5 (0.2 - 1.4) | 0.210 | 0.3 (0.1 - 1.8) | 0.207 |  |  |
|  | **Chronic GvHD** | | **Chronic GvHD** | | **Chronic GvHD** | |
|  | n = 140, events = 48 | | n = 140, events = 48 | | n = 140, events = 48 | |
|  | HR (95% CI) | p | HR (95% CI) | p | HR (95% CI) | p |
| ***CD19+ B cells*** |  |  |  |  |  |  |
| **> 13.1%** | 0.3 (0.1 - 0.8) | 0.019 |  |  |  |  |
| ***CD4+ T cells*** |  |  |  |  |  |  |
| **> 49.3%** |  |  | 1.7 1.0 - 3.1) | 0.057 |  |  |
| ***Combined*** |  |  |  |  |  |  |
| **CD19+^high^/CD4+^low^** |  |  |  |  | 0.8 (0.6 - 1.2) | 0.331 |
| Mismatch Donor | 2.6 (1.0 - 6.6) | 0.042 | 1.9 0.8 - 4.7) | 0.151 | 1.8 (0.8 - 4.4) | 0.183 |
| ATG vs. no ATG | 0.5 (0.1 - 1.7) | 0.251 | 0.6 0.2 - 2.1) | 0.404 | 0.6 (0.2 - 2.2) | 0.460 |

**Supplementary Table 4**: Reduced multivariate analysis

Reduced multivariate analysis of statistically significant lymphocyte subpopulations for investigating potential overfitting of the cox regression models. ATG = anti-thymocyte globulin, CI = confidence interval, CR = complete response, HR = hazard ratio, HSCT = hematopoietic stem cell transplantation, MAC = myeloablative conditioning, NRM= non-relapse mortality, OS = overall survival, RIC = reduced intensity conditioning

**Supplementary Figure 1**: Gating strategy for lymphocyte subpopulations

**Supplementary Figure 2:** ROC-curves for cut-off determination (NRM)

ROC-curves of all lymphocyte subpopulations regarding non-relapse mortality (NRM) within the first 100 days after allogeneic stem cell transplantation (HSCT).

**Supplementary Figure 3:** ROC-curves for cut-off determination (CMV infection)

ROC-curves for CD3^+^ T cells and CD3^+^CD8^+^ T cells regarding cytomegalovirus (CMV) infection within the first year after allogeneic stem cell transplantation (HSCT).

**Supplementary Figure 4:** Correlation between pre-transplant bone marrow NK cell and CD3⁺CD4⁻CD8⁻ T cell percentages. Correlation was assessed using Spearman's rank correlation (ρ = −0.075, *p* = 0.530).

**Supplementary Figure 5:** ROC-curves for cut-off determination (aGvHD)

ROC-curves for NK cells and CD3^+^CD4^-^CD8^-^ T cells regarding acute graft-versus-host disease (aGvHD).

**Supplementary Figure 6:** Correlation between pre-transplant bone marrow C19^+^ B cell and CD3⁺CD4^+^ T cell percentages. Correlation was assessed using Spearman's rank correlation (ρ = −0.499, *p* < 0.001).

**Supplementary Figure 7:** ROC-curves for cut-off determination (cGvHD)

ROC-curves for CD19^+^ B cells and CD3^+^CD4^+^ T cells regarding chronic graft-versus-host disease (cGvHD).
